# Supplementary material for: COVID-19 Pandemic and Helsinki University Hospital Personnel Psychological Well-Being: Six-Month Follow-Up Results
Source: Int J Environ Res Public Health. 2021 Mar 4;18(5):2524. doi: 10.3390/ijerph18052524 (PMC7967532; doi:10.3390/ijerph18052524)
Supplement: Supplementary file 1 [file ijerph-18-02524-s001.pdf]

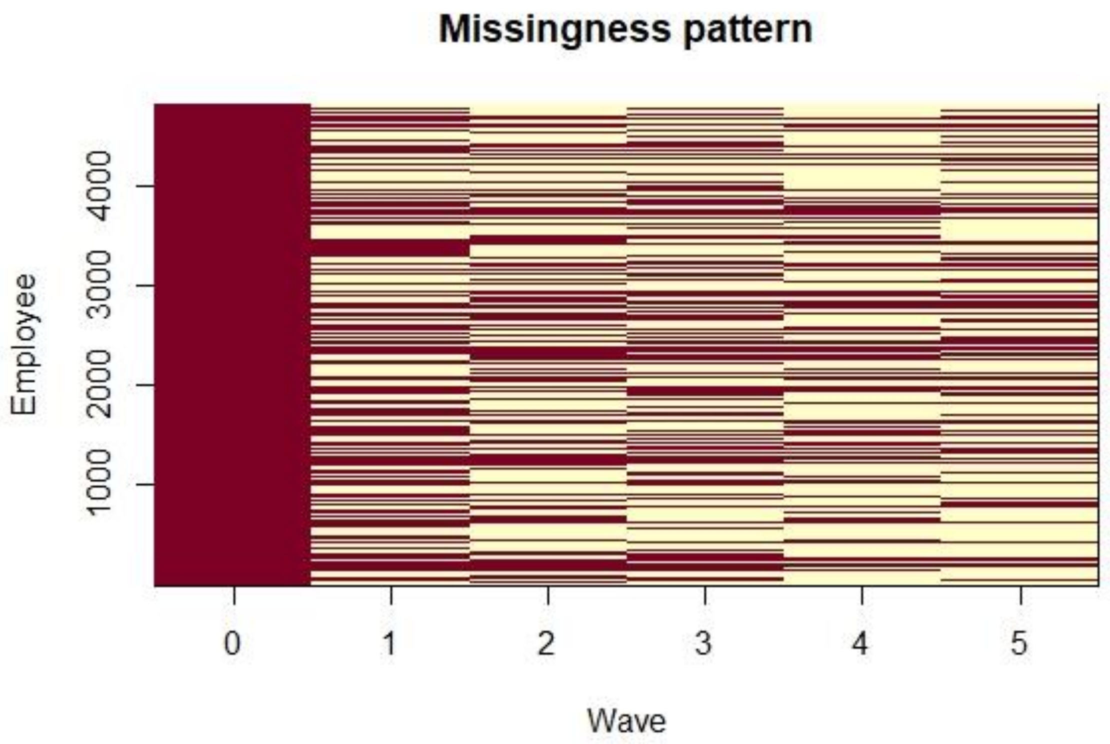

Figure S1. presents the missing data pattern in each survey wave (0–5).

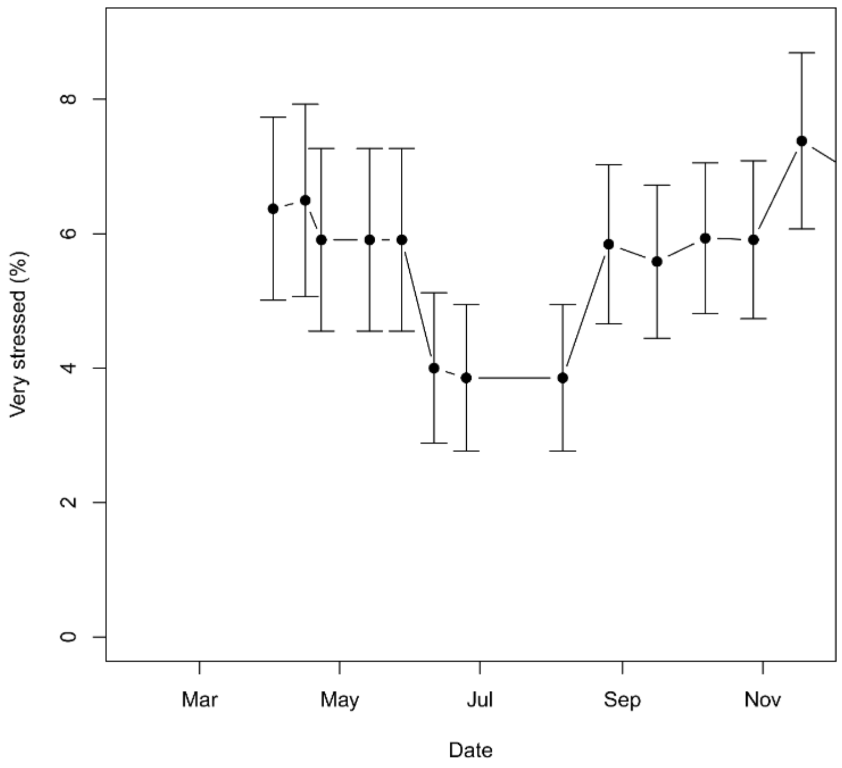

Figure S2. presents Finnish general population self-reported distress at the time of the study.
